# Submicron immunoglobulin particles exhibit FcγRII-dependent toxicity linked to autophagy in TNFα-stimulated endothelial cells

**DOI:** 10.1007/s00018-024-05342-9

**Published:** 2024-08-30

**Authors:** Wanida C. Hollis, Sehrish Farooq, M. Reza Khoshi, Mehulkumar Patel, Elena Karnaukhova, Nancy Eller, Karel Holada, Dorothy E. Scott, Jan Simak

**Affiliations:** 1 Center for Biologics Evaluation and Research, FDA, Silver Spring, MD USA; 2 Center for Devices and Radiological Health, FDA, Silver Spring, MD USA; 3 Institute of Immunology and Microbiology, First Faculty of Medicine, Charles University, Prague, Czech Republic; 4 Laboratory of Cellular Hematology, Division of Blood Components and Devices, Center for Biologics Evaluation and Research, Food and Drug Administration, OBRR, 10903 New Hampshire Avenue, WO Bldg. 52/72, Rm. 4210, Silver Spring, MD USA

**Keywords:** Protein aggregates, Immunogenicity, Vessel wall, Vascular toxicity, Protein corona, FcγR, Membrane microparticles, Biologics, Contaminants

## Abstract

In intravenous immunoglobulins (IVIG), and some other immunoglobulin products, protein particles have been implicated in adverse events. Role and mechanisms of immunoglobulin particles in vascular adverse effects of blood components and manufactured biologics have not been elucidated. We have developed a model of spherical silica microparticles (SiMPs) of distinct sizes 200–2000 nm coated with different IVIG- or albumin (HSA)-coronas and investigated their effects on cultured human umbilical vein endothelial cells (HUVEC). IVIG products (1–20 mg/mL), bare SiMPs or SiMPs with IVIG-corona, did not display significant toxicity to unstimulated HUVEC. In contrast, in TNFα-stimulated HUVEC, IVIG-SiMPs induced decrease of HUVEC viability compared to HSA-SiMPs, while no toxicity of soluble IVIG was observed. 200 nm IVIG-SiMPs after 24 h treatment further increased ICAM1 (intercellular adhesion molecule 1) and tissue factor surface expression, apoptosis, mammalian target of rapamacin (mTOR)-dependent activation of autophagy, and release of extracellular vesicles, positive for mitophagy markers. Toxic effects of IVIG-SiMPs were most prominent for 200 nm SiMPs and decreased with larger SiMP size. Using blocking antibodies, toxicity of IVIG-SiMPs was found dependent on FcγRII receptor expression on HUVEC, which increased after TNFα-stimulation. Similar results were observed with different IVIG products and research grade IgG preparations. In conclusion, submicron particles with immunoglobulin corona induced size-dependent toxicity in TNFα-stimulated HUVEC via FcγRII receptors, associated with apoptosis and mTOR-dependent activation of autophagy. Testing of IVIG toxicity in endothelial cells prestimulated with proinflammatory cytokines is relevant to clinical conditions. Our results warrant further studies on endothelial toxicity of sub-visible immunoglobulin particles.

**Supplementary Information:**

The online version contains supplementary material available at 10.1007/s00018-024-05342-9.

## Introduction

Immunoglobulins may form aggregates in blood components, plasma derivatives and purified monoclonal and polyclonal immunoglobulin (Ig) products. Protein particles in immunoglobulin products are of particular concern regarding potential effects on safety and efficacy. Intravenous Immune Globulins (IVIG) are manufactured from pooled plasma from thousands of healthy donors and containing at least 90% IgG, but also IgA and IgM. IVIG are used to treat primary and secondary immune deficiencies and autoimmune disorders, including certain neurological diseases, Kawasaki disease, and dermatomyositis. Although generally considered to be safe, therapy with IVIG and other immunoglobulin products has been associated with a wide spectrum of adverse events, including infusion-related phlogistic reactions, allergic responses, hemolysis, aseptic meningitis and vascular adverse events, such as myocardial infarction or stroke [1–7]. It has been shown that subvisible immunoglobulin particles can activate complement and innate immune cells and thus increase immunogenicity of IVIG and therapeutic monoclonal antibody products [8]. Little is known about direct effects of subvisible immunoglobulin particles on vascular endothelial cells, and no data are available on endothelial effects of immunoglobulin particles in the submicron size range.

Proteins in biologics readily agglomerate to form oligomers that evolve into protein aggregates, also called protein particles, of a wide range of size, shapes and other characteristics, including reversibility, conformation, chemical modifications, and morphology [9]. Protein aggregation can occur via self-association of monomers in their native or partially unfolded forms, in which aggregates are formed by colloidal interactions with minimal structural change, the self-association of non-native proteins through formation of unfolded or partially unfolded intermediates, or covalent reactions of native or structurally perturbed monomers [10–12]. Limitations and variations of different analytical methods further complicate aggregate characterization. Particle size is the most commonly used descriptor of protein particles. These are classified by size range: nanometer aggregates (< 100 nm, oligomers); submicron aggregates (100–1000 nm); micron aggregates (1–100 μm, subvisible particles); and aggregates greater than 100 μm (visible particles) [9]. These size categories are not consensually strict, the protein particle size range data in each study are strongly dependent on the analytical methods, laboratory expertise and instrument setting [13, 14]. Results may also be unintentionally biased by investigator’s focus of interest or choice of methodologies.

Protein aggregation can be induced by physical and chemical factors and may occur during product manufacturing, storage, transport, or other manipulations, such as product infusion. Physical factors promoting aggregation include shear stress, temperature changes (heating, cooling, freezing/thawing) or light exposure. Chemically related aggregation may occur with certain formulations variations (ionic strength, pH, excipient concentrations), or in the presence of divalent cations, vial or stopper leachables or exogenous contaminants. In addition, protein aggregation can be facilitated by different interphases exposed to protein solution, such as liquid/gas, liquid/solid, or liquid/liquid [15, 16].

One mechanism by which protein particles may form is by adsorption to nano- and microparticulate contaminants derived from several sources [17]. Some of the most important particulate contaminants are stainless steel nanoparticles shed from filling pumps [18], and glass nanoparticles and microparticles originating from glass vials or syringes [19]. Silicon oil, and rubber particles are also potent triggers of protein aggregation [20, 21].

Protein particles formed in biologics and other protein therapeutic products have a very broad size distribution and various shapes. While most studies are focused on visible aggregates and large subvisible particles, fewer studies have investigated protein particles in the submicron range.

The presence of protein particles has been associated with increased immunogenicity of protein therapeutic products [22–25]. Product immunogenicity resulting in humoral and cellular immune responses can affect product safety and efficacy profiles. Besides possible development of neutralizing antibodies to therapeutic proteins or cross-reactive antibodies to host’s endogenous proteins, immunogenicity may lead to activation of local or systemic complex inflammatory responses with auto-aggressive outcome of organ dysfunction manifested in various adverse events [26].

Subvisible protein particles present in protein therapeutics and other biologics may cause direct toxicity in the host tissues and/or induce immune responses affecting safety and efficacy of the product. Although several in vitro and in vivo studies document activities of protein particles on host immune cells and other tissues, it has been difficult to prove a causality between observed clinical adverse events and specific protein particle content in biologics investigation. It is not clear, which populations of particles characterized by size range, shape, composition, and surface characteristics, may be of greatest risk of causing immunogenicity or other clinically significant adverse events in patients. More detailed biological characterization of protein particles’ structure/function necessitates a system in which single physical or chemical parameter may be changed independently. For this purpose, we developed a model of different protein coronas presented on size-specific engineered spherical particles to investigate the effects of submicron size-specific particles on cultured endothelial cells.

## Materials and methods

### Silica microparticles (SiMPs)

Silica SiO_2_ microparticles 200 nm C-SIOS-0.200 (147040-10), 400 nm C-SIOS-0.400 (147070-10), 1000 nm C-SIOS-1.00 (117110-10), 2000 nm C-SIOS-2.00 (147-114-10), labeled as Silica SiO_2_ size standards-Nanospheres by the manufacturer, were from Corpuscular. DiagNano™ Blue Fluorescent Silica Nanoparticles, 200 nm (DNG-L090) were from CD Bioparticles. Submicron SiMPs were tested for endotoxin using Endosafe nexgen PTS with Charles River Laboratories LAL Test Cartridges (PTS55F). Endotoxin concentration in 200–1000 nm SiMPs at max concentrations used in the study (100 µg/ml) was < 0.02 EU/mL.

### Reagents

Licensed 10% IVIG products, Gammunex C and Privigen, were purchased from a distributor. IgG (14506) was from Millipore Sigma, human serum albumin (HSA, 2101) from InVitroCare, and human TNF-alpha (10602-HNAE) from Sino Biological. Antibodies used in the study: Anti p70S6 kinase (49D7) (2708), anti p-p70S6 kinase (T389) (9205), anti-rabbit IgG, HRP-conjugated (7074), anti-mouse IgG, HRP-conjugated (7076), beta-Tubulin (2148), and mTOR antibody (2972) were from Cell Signaling, TOM20 (F-10) Alexa Fluor 488 Ab (sc-177664), CD16 Ab (DJ130c) (sc-20052), CD64 Ab (10.1) (sc-1184), and CD32-B Ab (F-4) (sc-365864), CD32-A/B/C Ab (B-4) (sc-166711) were from Santa Cruz Biotechnology. Mouse IgG1 kappa isotype control (14-4714-82), CD64 mAb (10.1) (16-0649-81), CD32 mAb (6C4) (16-0329-81), CD16 mAb (EBIOCB16) (16-0168-85), were from Thermo Fisher Scientific. Anti SQSTM1/p62 Ab (EPR4844) was from Abcam.

### Cells

Human umbilical vein endothelial cells (HUVECs) were purchased from Lonza (CC-2519), and cultured in endothelial cells growth medium-2 (EGM-2) containing 2% FBS and growth factors supplement (CC-4176) [27]. For passaging the cells, the medium was removed, and the cells were washed with phosphate-buffered saline (PBS) (Thermo Fisher Scientific) and detached by 5 min incubation with Accutase (00-4555-56, Thermo Fisher Scientific) at 37 ^o^C. The cells were expanded to passage 3 and cryopreserved at 1 million cells per vial. Only passage 2–4 were used in the study.

### Preparation and characterization of immunoglobulin particles

The equilibrium adsorption of immunoglobulin onto SiMPs was carried out by incubating 100 µg/ml suspension of SiMPs with a concentration of 250 µg/ml IVIG for 24 h at 4 °C on a mini tube rotator (260750, Boekel Scientific), speed 6 orbits/min. The IVIG-SiMPs particles were sedimented by centrifugation at 20,000 x g for 30 min at 4 °C and washed once with PBS to remove the excess unbound protein. The estimated size, shape, dispersity, and average hydrodynamic diameter were assessed by scanning electron microscopy (SEM), nanoparticle tracking analysis (NTA) and flow cytometry as described in the Supplementary Information file.

### Circular dichroism (CD) analysis

The CD analysis was used to determine the secondary structure and folding properties of the immunoglobulin/albumin corona on SiMPs (IVIG/HSA-SiMPs) in comparison to the controls of SiMP and soluble protein samples. CD spectra were recorded on the Jasco J-815 Spectropolarimeter (Jasco) at the temperature 25 ± 0.2 ^o^C maintained by a Peltier thermostat (Jasco). Far- and near-UV CD spectra were measured for the samples in 10 mM PBS, pH 7.4 within wavelength ranges of 200–260 nm and 250–350 nm, respectively, in triplicate using a 2 mm pathlength quartz cuvette. The measurements were conducted with a bandwidth of 1.0 nm, a resolution of 0.2 nm and a scan speed of 100 nm/min. For the secondary structure evaluation, the samples were transferred from PBS into a CD-recommended buffer, 10 mM potassium phosphate, 100 mM (NH4)_2_SO_4_ buffer, further referred to as PBA, using Amicon 3 K filtration devices, and the far-UV CD spectra were recorded between 180 and 260 nm under the same conditions as listed above. The baseline was subtracted running a buffer, PBS or PBA, respectively, as a blank. The unsmoothed CD spectra of the samples were analysed for the secondary structural elements using the CDPro/CONTIN software (SP43).

### Assessment of cell viability and cytotoxicity

HUVEC cells were cultured in EGM-2 containing 2% FBS along with supplements with or without tumor necrosis factor-alpha (TNFα) followed by addition of IVIG-SiMPs or IgG-SiMPs, or HSA-SiMPs for 24 h. The viability of the cells was assayed using Cell Counting Kit-8 (CCK-8) and lactate dehydrogenase (LDH) kits according to the manufacturers protocols, as described previously [28].

### Apoptosis and cell cycle analysis

HUVECs were cultured in 6 well plates to approximately 80% confluence and exposed to IVIG-SiMPs or HSA-SiMPs or SiMPs for 24 h at 37 °C with 5% CO_2_. Subsequently, the cells were harvested and stained with lactadherin/propidium iodide (PI) (Thermo Fisher Scientific) for apoptosis analysis and with Alexa Fluor™ 488-conjugated anti-BrdU mAb and PI using the APO-BrdU™ TUNEL Assay Kit (Thermo Fisher Scientific) for TUNEL-cell cycle analysis, as described previously [28].

### Monitoring autophagy

For autophagosome detection, HUVECs were transfected with BacMam PremoTM Autophagy Sensor LC3B-GFP, as described in manufacturer’s protocol. Following transfection, the cells were treated with IVIG-SiMPs and the controls for 24 h. Afterwards, the cells were fixed, permeabilized, and stained for specific organelle staining markers before imaging with confocal microscope (LSM 700) (Zeiss Microscopy) [29, 27]. To quantify the autophagy-lysosome dependent degradation proteins (LC3, p62) and mTOR signaling related proteins (p/mTOR, p/p70SK), the IVIG-SiMPs and the control treated cells were harvested and lysed in RIPA buffer (Thermo Fisher Scientific) supplemented with protease inhibitor cocktail for SDS-PAGE and Western blot analysis. The bands were quantified by ImageJ software [30].

### Flow cytometry analysis of released extracellular vesicles (EVs)

HUVECs were treated with IVIG-SiMPs or controls for 24 h. and the released EVs were collected as described previously [28]. The EV samples were labelled with different antibodies/staining solutions, including PE-conjugated CD105 mAb, FITC-conjugated lactadherin, and Alexa 488- TOM20 mAb in the dark for 30 min at room temperature and subjected to flow cytometry analysis. The calibration of the LSR II flow cytometer was performed using CST (BD Bioscience) and ApogeeMix calibration beads (Apogee Corporation). The flow rate and count of EVs per µL of medium were evaluated by using TruCount™ beads (BD Bioscience). The data analysis was performed using FlowJo software.

### Detection and functional assays of FcγR

HUVECs were cultured and treated with TNFα for 24 h and the expression of FcγRI, II and III was evaluated by Western blot, LSCM and In-cell enzyme-linked immunosorbent assay (In-cell ELISA). For functional assay of FcγR, the cells were seeded into 96 well plates and subjected to preincubation with FcγR inhibitory antibodies or isotype controls for 2 h prior to treatment with IVIG-SiMPs, SiMPs and HSA-SiMPs for 24 h. Then the cell viability was assessed using the CCK-8 assay.

### Statistical analysis

All data were plotted using GraphPad Prism (version 10), analysed using one-way ANOVA followed by Tukey’s post hoc test, and presented as the mean ± SEM (standard error of mean), with p values < 0.05 were considered significant.

## Results

### Engineered particle protein corona model for investigation of size-dependent biological effects

**Fig. 1:**
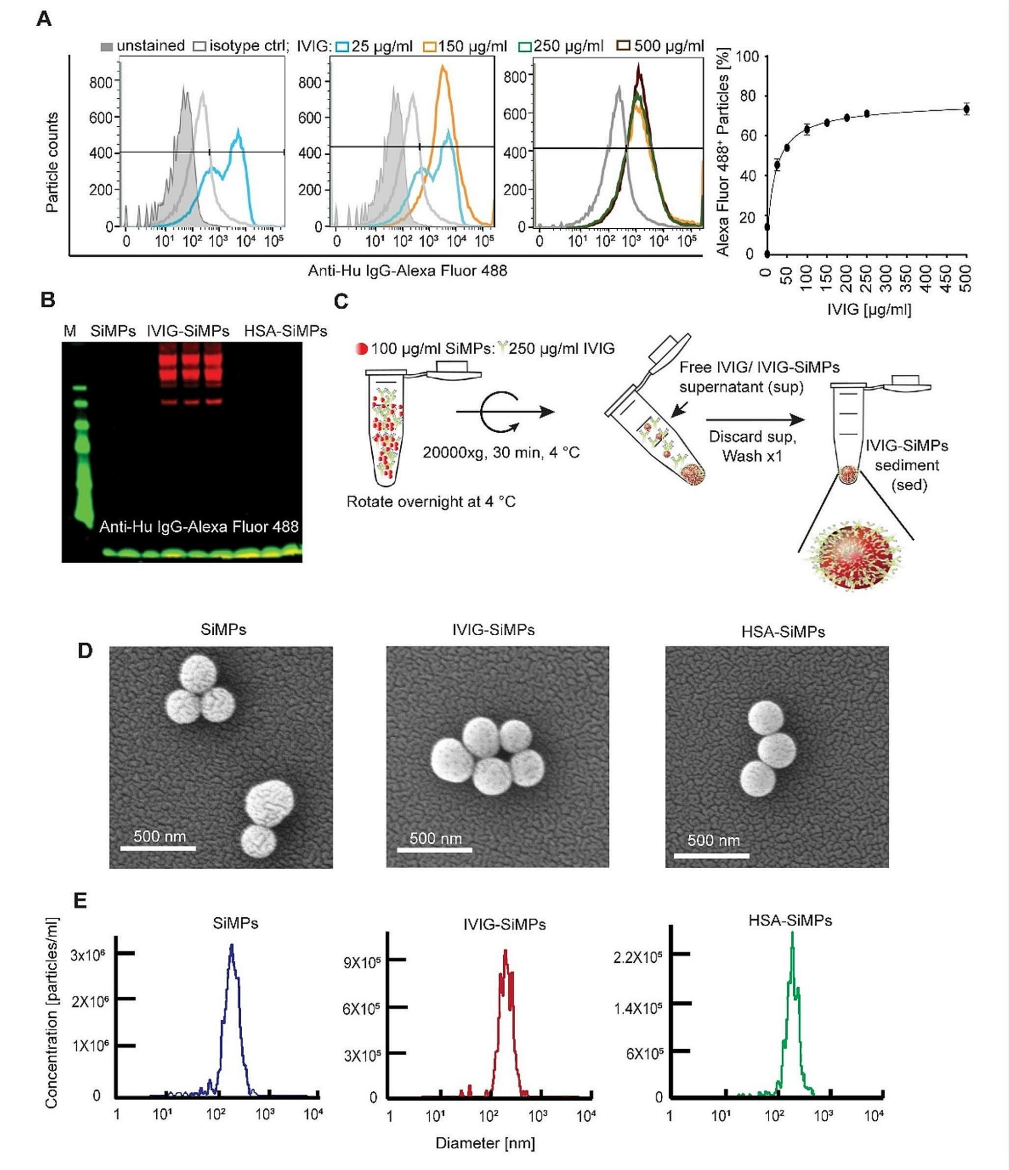
Preparation and characterization of IVIG-SiMPs. **(A)** SiMPs at 100 µg/ml were incubated overnight at 4 ^o^C with various concentrations of IVIG (0–500 µg/ml). The binding was detected with anti-human IgG conjugated Alexa 488 using **(A)** flow cytometry and **(B)** Western blot analysis. **(C)** Generation of IVIG protein corona on silica particles. IVIG-SiMPs were generated by gently mixing 250 µg/ml IVIG with 100 µg/mL silica particles on a rotator at 4 °C for 24 h. The resulting particles were subjected to **(D)** SEM and **(E)** NTA for evaluation of morphology and hydrodynamic size distribution of the particles. Abbreviations used: IVIG, intravenous immunoglobulin; SiMPs, silica microparticles, IVIG-or HSA-SiMPs, IVIG or HSA coated SiMPs; sed: sediment; sup, supernatant; ctrl, control

To investigate particle size-dependent adverse effects of immunoglobulin aggregates, we have developed a model of engineered spherical SiMPs of distinct sizes (200–2000 nm) coated with different IVIG-, purified IgG-, or albumin (HSA)-coronas and analysed their effects on cultured HUVEC. We employed flow cytometry to assess the optimum binding concentration of IVIG onto SiMPs. 200 nm SiMPs at 100 µg/ml were used to titrate the IVIG concentration across a range from 0 to 500 µg/ml. The result revealed that the binding of IVIG on SiMPs reached its maximum around 150–250 µg/ml **(**Fig. 1A**)**. Western blot analysis further validated the specificity of IVIG binding to particles and no IgG was detected with anti-human IgG Alexa fluor 488 on HSA-SiMPs, excluding any possibility of the HSA contamination with IgG **(**Fig. 1B**)**.

The 250 µg/ml IVIG/ 100 µg/ml SiMPs concentration ratio was selected for the subsequent experiment in the preparation of IVIG-SiMP particles, as depicted in Fig. 1C. SEM **(**Fig. 1D**)** was used to characterize the size and morphology of 200 nm IVIG-SiMPs, control bare SiMPs, and HSA-SiMPs. The results demonstrated a spherical morphology, with size distribution consistent with values reported by the manufacturer. NTA revealed narrow size distribution of the bare and protein coated SiMPs (Fig. 1E**).** The increase in hydrodynamic diameter of IVIG-SiMPs and HSA-SiMPs compared to bare SiMPs reflected formation of relatively uniform and hard protein corona (Supplementary Table T1).

### Circular dichroism (CD) reveals no significant impact of SiMPs on secondary structure of immunoglobulin and albumin proteins

**Fig. 2:**
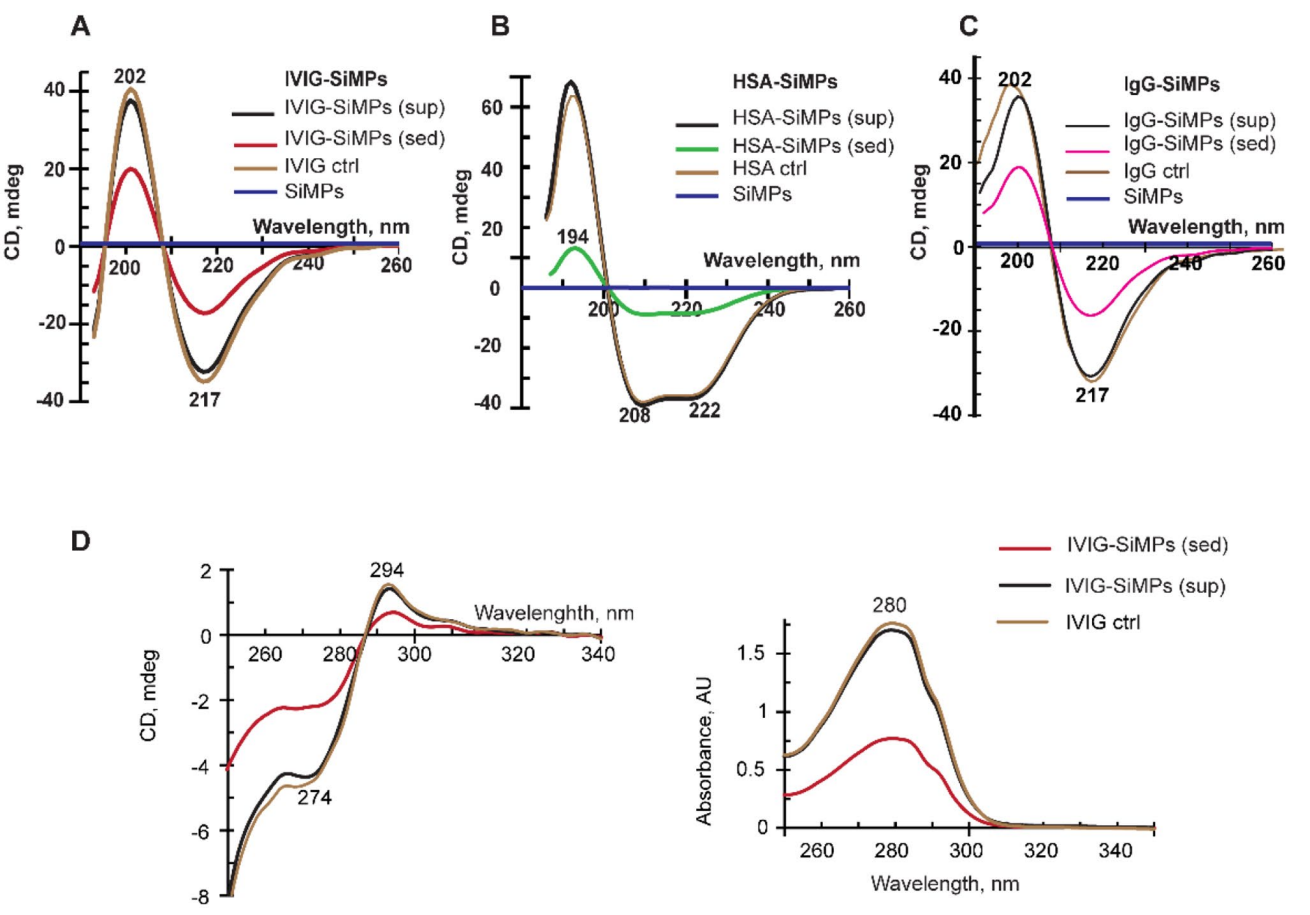
Impact of SiMPs on secondary structure of immunoglobulin and albumin proteins. Far-UV CD spectra were measured to evaluate the secondary structure of **(A)** IVIG-SiMPs, **(B)** HSA-SiMPs, and **(C)** IgG-SiMPs samples in comparison with their respective control proteins IVIG and HSA. **(D)** Near-UV CD data (**left**) and complementary absorbance spectra (**right**) of IVIG-SiMPs samples shown versus control IVIG. Abbreviation used: sup, supernatant; sed, sediment; ctrl, control

CD spectroscopy was utilized to monitor conformational changes in IVIG, purified IgG, and HSA per their binding to SiMPs in comparison with their respective control (uncoated) protein samples. According to the far-UV CD spectra (Fig. 2**)**, SiMPs per se show no significant optical activity (blue trace near zero-line). The IVIG-SiMPs protein corona samples obtained from both the supernatant and sediment samples exhibit characteristic far-UV CD spectrum (Fig. 2A; red trace corresponds to the sediment) with two extremums, negative at 217 nm and positive at 202 nm typical for beta-structure-rich proteins, which is essentially similar to the pattern of the control IVIG solution (brown trace). Very similar far-UV CD data were collected for HSA-SiMPs protein corona and control HSA solution sample (shown in Fig. 2B**)**, as well as in case of purified IgG-SiMPs and control IgG samples **(**Fig. 2C**).** Calculations of the secondary structure elements (in percent) further demonstrated only small insignificant differences between the SiMPs-associated proteins and their control uncoated counterparts (Supplementary Table T2 Possible impact of SiMPs on protein tertiary structure was evaluated by the near-UV CD spectra, as shown for IVIG-SiMPs and control IVIG (Fig. 2D). Overall, our CD data indicated that the formation of a protein corona of IVIG and HSA on SiMPs had no significant impact on the secondary or tertiary structures of IVIG and HSA, thus suggesting that the functional properties of IVIG and HSA-SiMPs were preserved.

### Submicron IVIG-SiMPs caused particle size-dependent toxicity in TNFα-stimulated HUVECs

**Fig. 3:**
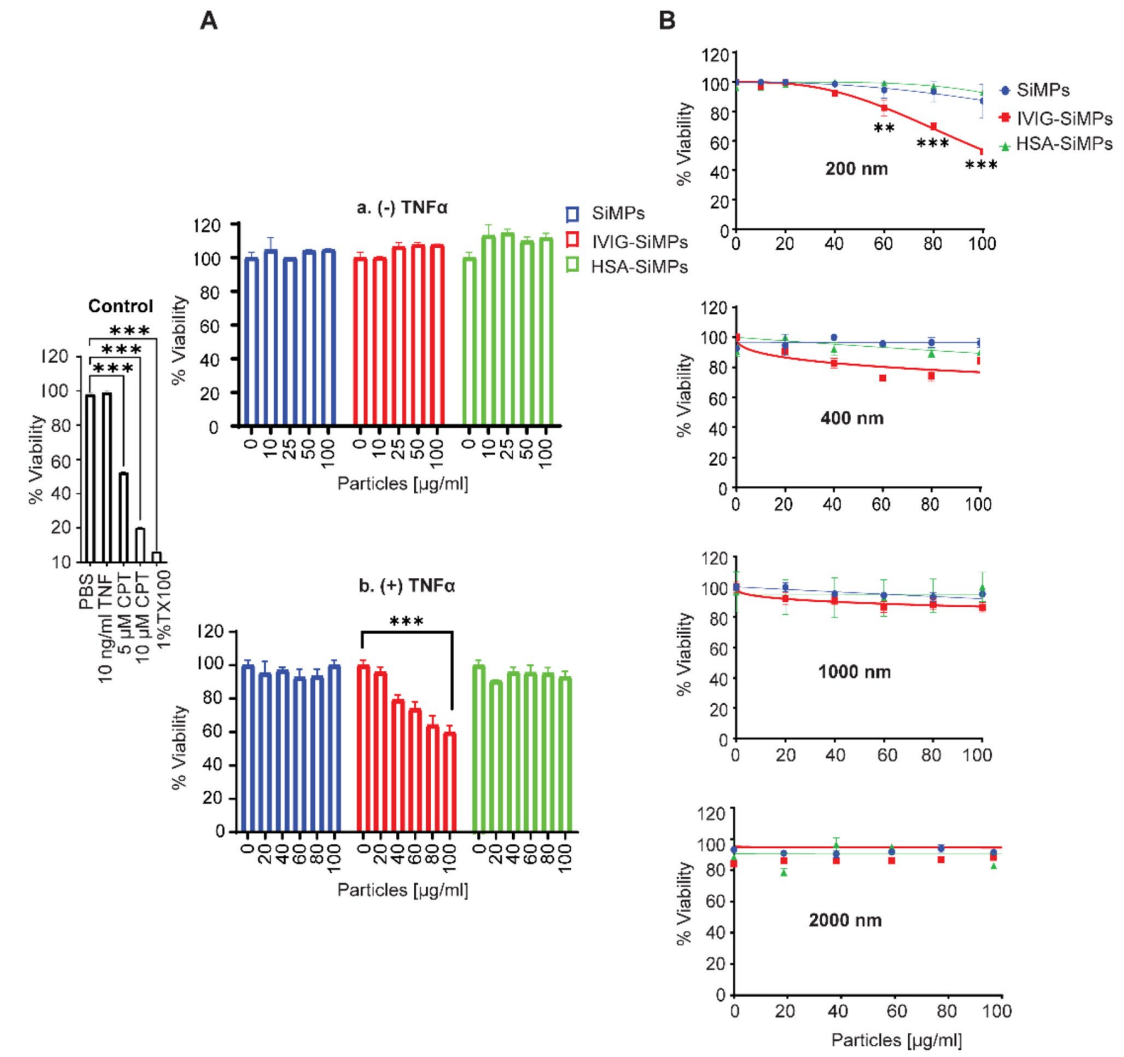
Size dependent toxicity of IVIG-SiMPs in TNFα-stimulated HUVECs. **(A)** HUVECs were exposed to 0–100 µg/mL of 200 nm SiMPs (blue), IVIG-SiMPs (red), and HSA-SiMPs (green) for 24 h. The cells were treated without **(A, a)**, and with **(A, b)** 10 ng/ml TNFα compared to positive controls 5 or 10 µM camptothecin (CPT) and 1% Triton X100 (TX100). Cell viability was evaluated using the CCK-8 assay. **(B)** Viability of TNFα-stimulated HUVECs after 24 h. treatment with different sizes (200, 400, 1000, and 2000 nm) of IVIG-SiMPs compared with the controls SiMPs and HSA-SiMPs. Data are presented as the percentage of cell viability compared to medium-treated HUVECs. Mean ± SEM, *n* = 3, ** *p* < 0.01, *** *p* < 0.001

The size-dependent toxicity of IVIG-SiMPs was assessed using cell viability (CCK-8) and membrane integrity (LDH leakage) assay. Different sizes of IVIG-SiMPs were exposed to HUVECs for 24 h. No significant toxicity was observed in unstimulated HUVECs exposed to IVIG-SiMPs, HSA-SiMPs or bare SiMPs in the size range from 200 to 2000 nm (Fig. 3A, a). In contrast, in HUVEC stimulated with a low concentration of TNFα (10 ng/ml), treatment with 200 nm IVIG-SiMPs resulted in a dose-dependent decrease in cell viability (Fig. 3A, b). No significant changes of cell viability were observed after treatment with corresponding concentrations of HSA-SiMPs or bare SiMPs. The toxicity of IVIG-SiMPs was most prominent for 200 nm SiMPs and decreased with larger particle size, as demonstrated by both CCK-8 (Fig. 3B) and LDH (Supplementary Fig. S1A) assays. These results were confirmed with two different commercial IVIG products and a purified research grade IgG (Supplementary Fig. S1B). Similar effects occurred in LPS-stimulated HUVECs exposed to IVIG-SiMPs (Supplementary Fig. S1C). Bare SiMPs in the size range 200–2000 nm at concentrations up to 100 µg/ml (Supplementary Fig. S1D), as well as soluble IVIG, IgG, or HSA proteins at concentrations up to 5 mg/mL (Supplementary Fig. S1E) did not cause any significant effects on unstimulated or TNFα-stimulated HUVECs. Submicron IVIG-SiMPs augmented proinflammatory activation of TNFα-stimulated HUVECs. Immuno-flow cytometry showed that in HUVEC stimulated with very low concentration of TNFα (1–5 ng/ml), 200 nm IVIG-SiMPs caused an additive increase in cell surface expression of ICAM1 and tissue factor (TF) (Supplementary Figure S2, A-B) and increased release of ICAM1^+^ and TF^+^ EVs (Supplementary Figure S2, C-D), indicating potentiation of proinflammatory stimulation of the cells.

### Submicron IVIG-SiMPs induced cell cycle arrest and apoptosis in TNFα-stimulated HUVECs

**Fig. 4:**
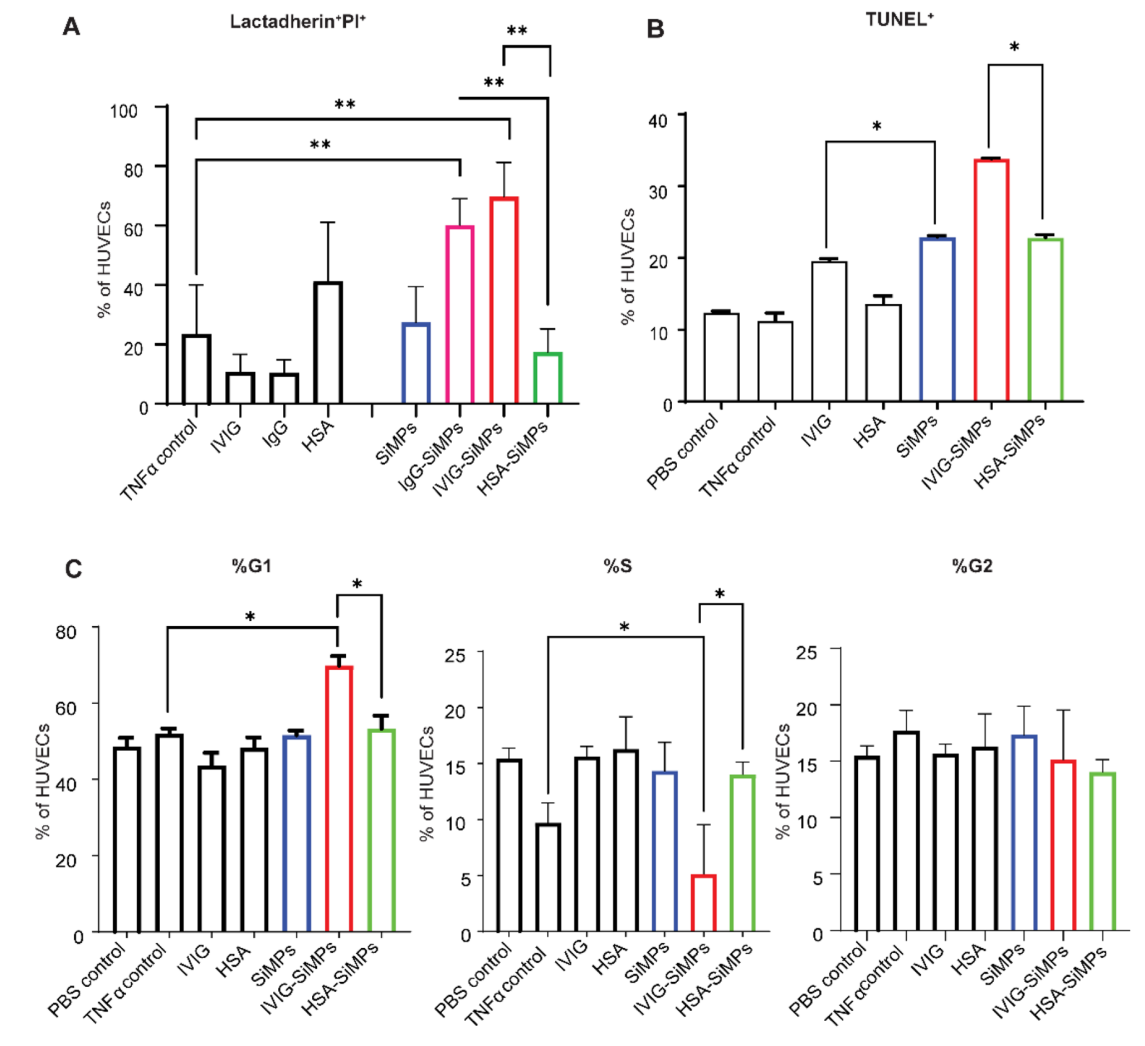
200 nm IVIG-SiMPs induced *G*1 arrest and apoptosis in TNFα- stimulated HUVECs. **(A)** IVIG-SiMPs, as well as IgG-SiMPs (80 µg/ml) induce apoptosis in TNFα-stimulated HUVECs after 24 h. treatment, as detected by lactadherin/PI assay. **(B)** The apoptosis indicated by the increase in the TUNEL-positive (TUNEL^+^) cell population and **(C)** G1 cell cycle arrest in TNFα-stimulated HUVECs after 24 h. of treatment. The cell cycle analysis was performed by flow cytometry using the APO-BrdU™ TUNEL Assay Kit. Mean ± SEM, *n* = 3, ** *p* < 0.01, *** *p* < 0.001

To investigate the mechanism underlying the cell death caused by submicron IVIG-SiMPs, apoptosis in TNFα-stimulated HUVECs was analyzed by lactadherin/PI and TUNEL assays. As shown in Fig. 4A, after 24 h treatment, 200 nm IVIG-SiMPs (80 µg/ml) or IgG-SiMPs caused marked increase in lactadherin^+^ PI^+^ cells, in contrast to the lack of effect in the presence of HSA-SiMPs, bare SiMPs, soluble IVIG, IgG, or HSA proteins. Corresponding increases in TUNEL^+^ cells (Fig. 4B) and a moderate G1 arrest in the presence of 200 nm IVIG-SiMPs treated HUVECs **(**Fig. 4C**)** was also observed in TNFα-treated HUVECs, whereas HSA-SiMPs, bare SiMPs and soluble proteins had slight to no significant effect on the cell cycle.

### Submicron IVIG-SiMPs induced mTOR-dependent activation of autophagy in TNFα-stimulated HUVECs

**Fig. 5:**
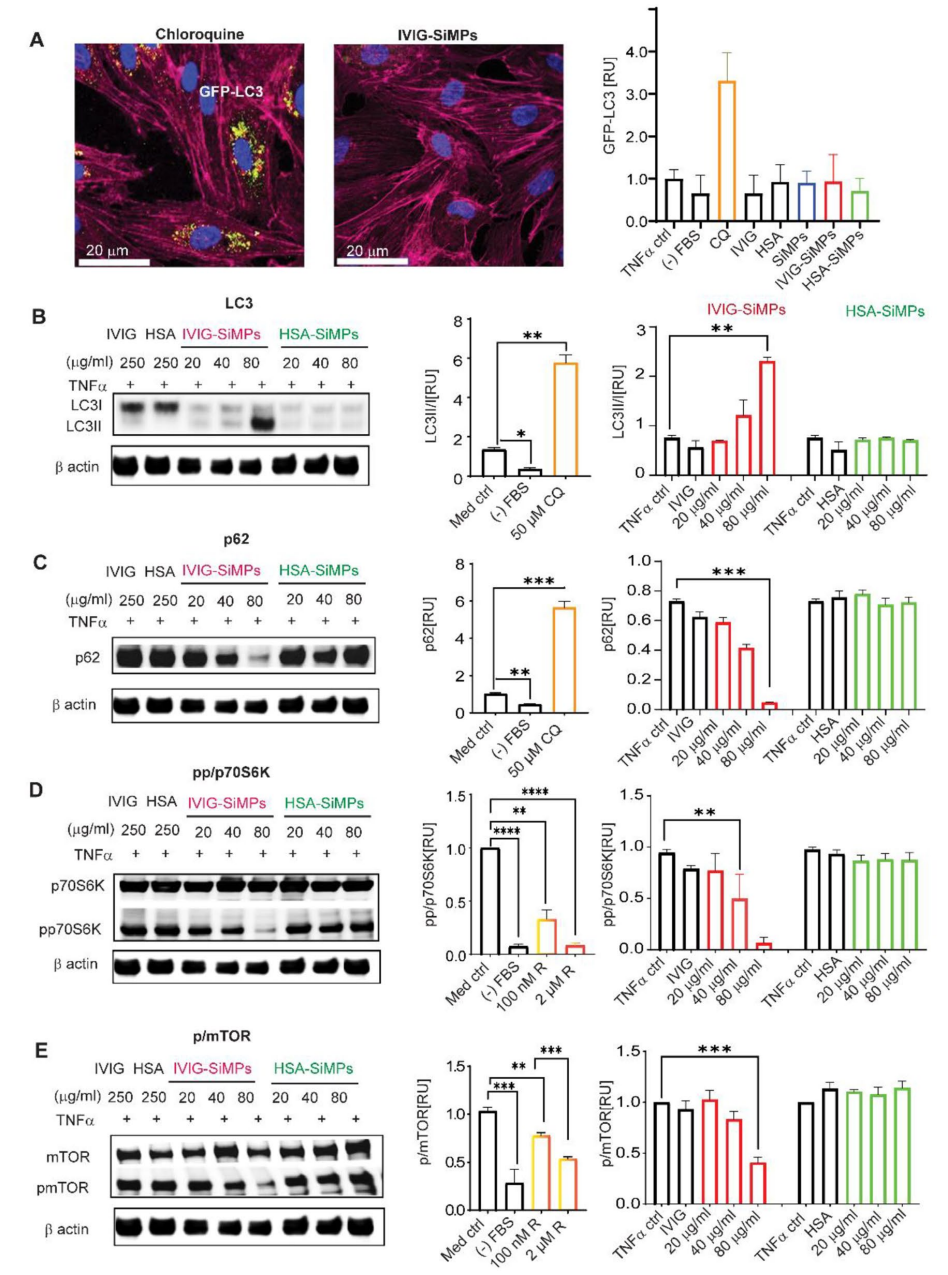
IVIG-SiMPs induced mTOR-dependent activation of autophagy in TNFα-stimulated HUVECs. **(A)** Analysis of autophagosome accumulation by laser scanning confocal microscopy (LSCM). HUVECs were transfected with GFP-LC3 for 24 h. at 37 ^o^C. Subsequently, the cells were treated for additional 24 h. with 100 µg/ml of 200 nm SiMPs, IVIG-SiMPs, or HSA-SiMPs in the presence of 10 ng/ml TNFα. 50 µM chloroquine (CQ) and serum starvation ((-) FBS) served as positive controls. GFP-LC3 fluorescent intensity was observed by LSCM and the intensity was quantified by ImageJ. The cells were washed with Hanks balanced salt solution (HBSS), fixed with 2% paraformaldehyde, and stained with CellMask™ Deep Red Actin Tracking (red) and Hoechst33342 (blue) for 60 min. The slides were mounted with gold antifade mounting media and imaged using a confocal microscope (LSM700) with a 40x oil objective. Scale bars represent 20 μm. Arrows show GFP-LC3 in HUVECs after the treatment. **(B)** the autophagy markers LC3, (**C)** p62, **(D)** p70S6k/pp70SK and **(E)** mTOR/pmTOR were assessed by Western blot analysis and the band intensities were quantified by ImageJ. HUVECs were treated for 24 h. with varying concentrations (0, 20, 40, 80 µg/ml) of 200 nm SiMPs, IVIG-SiMPs, and HSA-SiMPs in the presence of 10 ng/ml TNFα. 30 µM CQ, 100 nM Rapamycin (R) and serum starvation ((-) FBS) were included as controls. The ImageJ analysis was performed to quantitatively measure band intensities, with the band normalized to β-actin, which was derived from the same samples, and present the values as relative arbitrary units of phosphorylated over non-phosphorylated bands. Mean ± SEM, *n* = 3, * *p* < 0.05, ** *p* < 0.01

To investigate the role of autophagy following the internalization of IVIG- and HSA-SiMPs in TNFα-stimulated HUVECs, we utilized LC3-GFP transfected cells to visualize autophagosomes. Laser scanning confocal microscopy (LSCM) demonstrated autophagosome accumulation in cells treated with the autophagy flux inhibitor chloroquine, serving as a positive control. In contrast, HUVECs treated with 200 nm IVIG-SiMPs in the presence of 10 ng/ml TNFα exhibited only slightly elevated autophagosome levels **(**Fig. 5A**).** Western Blot analysis of the conversion of LC3I to LC3II showed LC3II significantly elevated as concentrations of IVIG-SiMPs were increased **(**Fig. 5B**).** Levels of p62 protein, which is preferentially degraded during autophagy, showed a dose dependent decrease indicating induction of autophagy by 200 nm IVIG-SiMPs treatment, similar to the serum starvation control **(**Fig. 5C**).** Observed p62 protein degradation suggests specific activation of autophagy by 200 nm IVIG-SiMPs rather than blockade of the autophagic flux. Moreover, we observed diminished phosphorylated forms of p70S6K **(**Fig. 5D**)** and mTOR **(**Fig. 5E**)** following 200 nm IVIG-SiMPs treatment, indicating mTOR-dependent activation of autophagy in TNFα-stimulated HUVECs.

### Submicron IVIG-SiMPs induced in TNFα-stimulated HUVECs a release of EVs positive for PS, autophagosome and mitochondrial markers

**Fig. 6:**
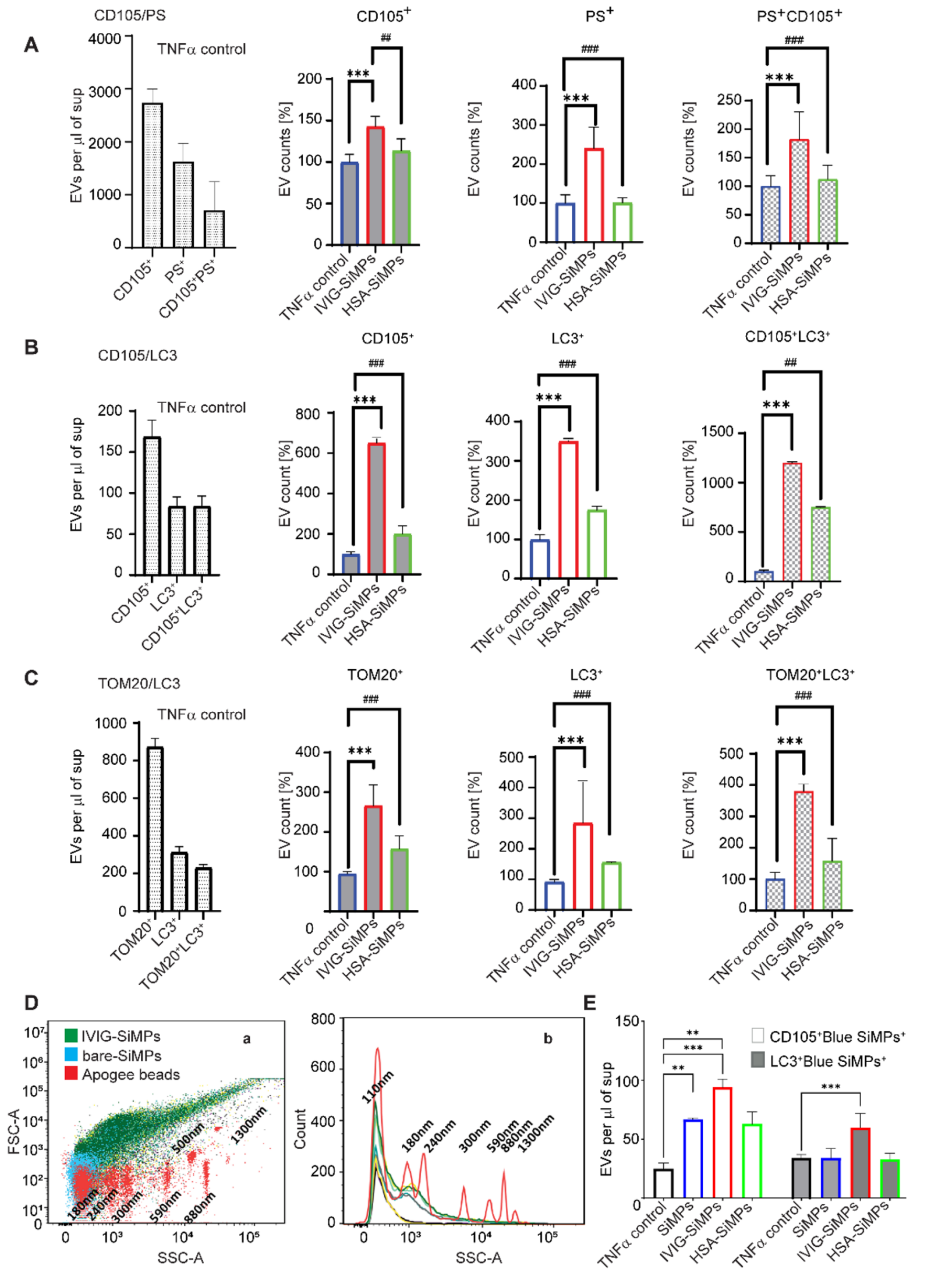
IVIG-SiMPs increased the release of EVs positive for autophagosome and mitochondrial markers in TNFα-stimulated HUVECs. After exposure HUVECs to 200 nm IVIG-SiMPs (100 µg/ml) and the controls for 24 h. in the presence of 10 ng/ml TNFα, the released of EVs in the cell culture supernatant were collected and subjected to flow cytometry for evaluation of the EV size distribution and phenotyping. The EV surface expression of the following markers was analyzed: **(A)** CD105/PS for endothelial cell origin and procoagulant phenotype, **(B)** CD105/LC3 to detect the autophagosome marker, and **(C)** TOM20/LC3 to detect EVs of mitochondrial origin and products of mitophagy. **(D)** Flow cytometry analysis of EV size distribution was performed in comparison to the ApogeeMix reference silica beads. The overlayer **(D, a)** pseudo dot plot and **(D, b)** histogram plot of IVIG-SiMPs, bare SiMPs, and Apogee beads. **(E)** HUVECs were treated with IVIG-coated 200 nm blue, fluorescent SiMPs (100 µg/ml) to monitor the release of EVs associated with IVIG-SiMPs and positive for endothelial membrane (CD105) and autophagosome (LC3) markers. Mean ± SEM, *n* = 3, *, # *p* < 0.05, **, ## *p* < 0.01, ***, ### *p* < 0.001

Flow cytometry was used to explore the impact of 200 nm IVIG-SiMPs treatment on the release of EVs from TNFα-stimulated HUVECs. As illustrated in Fig. 6, phenotyping of EVs using anti CD105 mAb and lactadherin **(**Fig. 6A**)** demonstrated significantly higher counts of CD105^+^PS^+^EVs in the cell culture medium of 200 nm IVIG-SiMPs treated HUVECs, compared to HSA-SiMPs treated cells and TNFα-stimulated control cells. CD105/LC3 labelling of EVs **(**Fig. 6B**)** revealed markedly higher counts of EVs positive for autophagosome marker LC3 in 200 nm IVIG-SiMPs-treated cultures. Association of IVIG-SiMPs with endothelial EVs (CD105^+^) and specifically with endothelial EVs of autophagosomal origin (CD105^+^LC3^+^) was demonstrated using cell treatment with IVIG-blue SiMPs, **(**Fig. 6E**).** Moreover, increase of endothelial EVs positive for both autophagosomal marker LC3 and a mitochondrial marker TOM20 suggests activation of mitophagy in TNFα-stimulated HUVECs after treatment with 200 nm IVIG-SiMPs (Fig. 6C**).** Furthermore, we employed flow cytometry analysis to estimate the size distribution of EVs **(**Fig. 6D**).** Detected EVs released from TNFα-stimulated HUVECs after treatment with 200 nm IVIG-SiMPs were in the size range of 100–1500 nm when compared to the size of Apogee Mix silica reference beads. Most of the analysed EV populations were small EVs < 300 nm and a low percentage of EVs was in the medium size range 300–500 nm, with less than 1% of EVs being larger than 500 nm.

### Cellular uptake and toxicity of submicron IVIG-SiMPs was dependent on FcγRII receptor expression on HUVECs, which increased after TNFα-stimulation

**Fig. 7:**
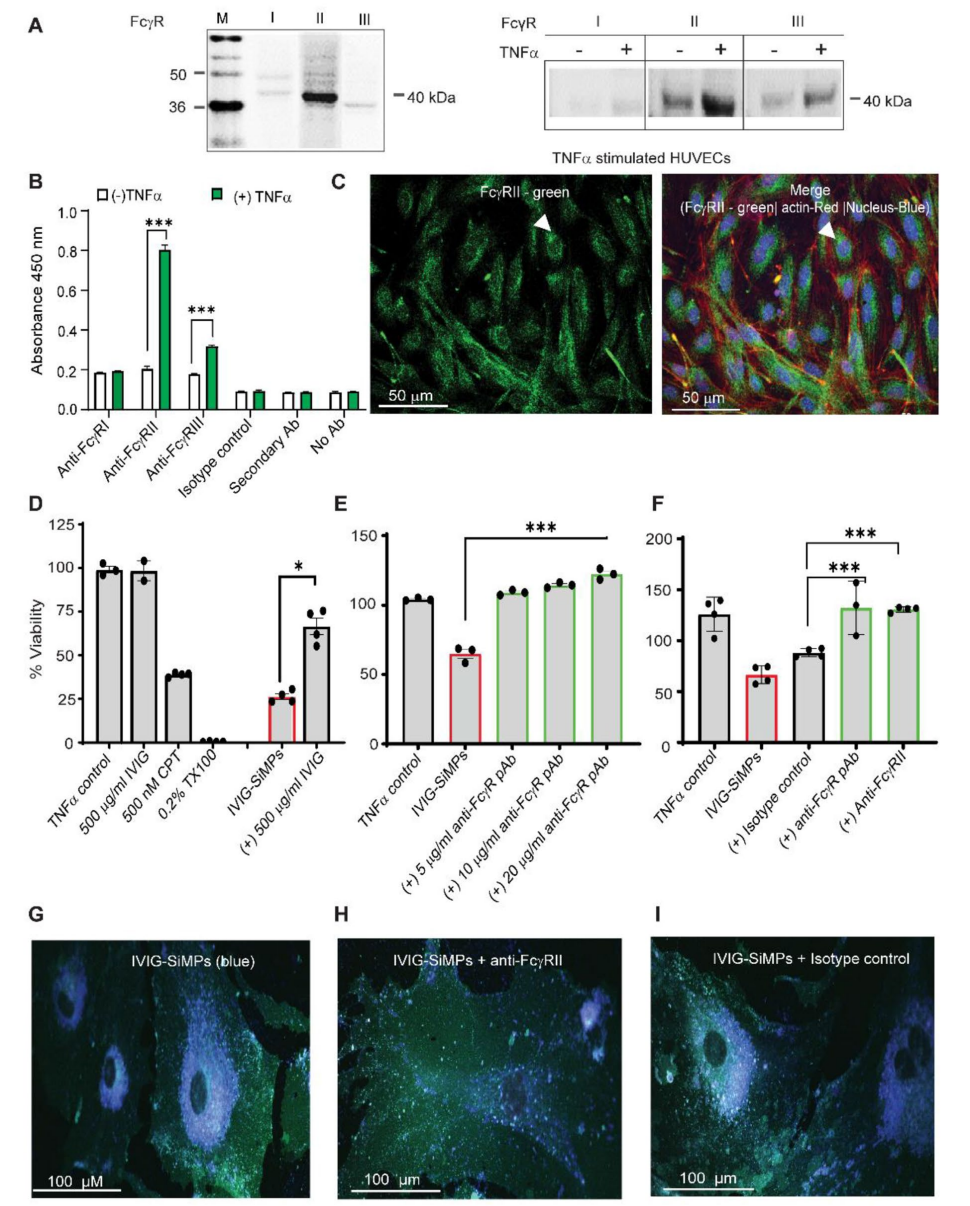
Cellular uptake and toxicity of 200 nm IVIG-SiMPs in TNFα-stimulated HUVECs is FcγRII dependent. FcγRII expression is increased in TNFα-stimulated HUVECs. The FcγR expression was evaluated by **(A)** Western blot, **(B)** ELISA, and **(C)** immunofluorescence (IF) cytostaining using FcγR specific antibodies. For IF staining, FITC conjugated FcγRII was utilized, along with nuclear staining (Hoechst 33342-blue), and actin tracking staining CellMask Deep Red. The slides were mounted using gold antifade mounting media (Invitrogen) and imaged using a confocal microscope (LSM700) with a 40x oil objective. Scale bars represent 50 μm. Arrows show FcγRII expressed in TNFα-stimulated HUVECs. **(D-F)** Soluble IVIG and anti-FcγR antibody markedly decreased the toxicity of 200 nm IVIG-SiMPs (100 µg/ml; 24 h.) in TNFα-stimulated HUVECs. TNFα -stimulated HUVECs were pre-incubated with **(D)** 500 µg/ml IVIG or **(E)** anti**-**FcγR pAb, or (**F**) anti-FcγRII mAb for 2 h. at 37 ^o^C. The cells were then incubated with IVIG-SiMPs for an additional 24 h. The percentage viability of HUVECs was measured by CCK-8 assay. The results are representative data of at least three independent experiments, *p < 0.05. **(F)** Pre-incubation of TNFα-stimulated HUVECs with anti-FcγRII mAb decreased the uptake of 200 nm IVIG-coated blue, fluorescent SiMPs assessed by LSCM. HUVECs were incubated with 200 nm IVIG-coated blue, fluorescent SiMPs (100 µg/ml) **(G)** without or pre-exposure with (**H)** 10 µg/ml anti-FcγgRII mAb or **(I)** the corresponding isotype control mAb. Then the cells were stained with cell mask green for 30 min at 37 ^o^C and subjected to LSCM analysis. Mean ± SEM, *n* = 3, * *p* < 0.05, ** *p* < 0.01, *** *p* < 0.001

To investigate a potential role of FcγRs in response to IVIG-SiMPs, we analyzed expression of FcγRs in HUVECs. The WB analysis showed almost no detection of FcγRI and low levels of FcγRII and FcγRIII in unstimulated HUVECs. In TNFα-stimulated HUVECs, the FcγRII expression markedly increased, and a smaller increase of FcγRIII expression was also observed (Fig. 7A**).** The WB results were in accord with the ELISA results (Fig. 7B**)**, where FcγRII expression increased ~ 6–8 folds (*P* < 0.0001) in TNF-stimulated HUVECs. Upregulation of FcγRII expression was TNFα concentration- and time-dependent (Supplementary Fig. S3 A-B). LSCM immunodetection confirmed the expression of FcγRII on the surface of the TNFα-stimulated HUVECs **(**Fig. 7C**).**

To investigate the role of FcγRII and FcγRIII in 200 nm IVIG-SiMPs cellular uptake, TNFα-stimulated HUVECs were treated with blue fluorescent 200 nm IVIG-SiMPs. The cells were stained with Cell Mask green (plasma membrane) and pink Syto dye (nucleus). Following a 24 h. treatment, the IVIG-SiMPs (identified by their blue color) were shown by LSCM to be internalized into HUVECs. Pretreatment of the cells with anti-FcγRII antibody (CD32 mAb) virtually abolished uptake of IVIG-SiMPs **(**Fig. 7H**).** No effect of a corresponding isotype control was observed (Fig. 7I). Pre-treatment of TNFα-stimulated HUVECs with an anti-FcγR polyclonal antibody **(**Fig. 7E**)** or anti-FcγRII mAb **(**Fig. 7F**)** significantly suppressed effect of 200 nm IVIG-SiMPs on the cell viability, indicating that the toxicity of submicron IVIG-SiMPs on TNFα-stimulated HUVECs is FcγRII dependent.

## Discussion

While some protein aggregates appear to be innocuous, others have been associated with serious adverse events in patients, such as immunogenicity and infusion reactions. Effects on the products include loss of potency and stability. A certain level of aggregation is inevitable in protein solutions, but it has been technically challenging to identify aggregate qualities that are associated with or predicted to have adverse consequences for patients. Formation of protein aggregates in therapeutic products, such as plasma derivatives, blood components and other biologics, is affected by various physical and chemical factors leading to a product-specific heterogenous spectrum of protein particles. Accelerated stress methods are used to model protein aggregation which occurs during manufacturing, storage, shipping, and administration of protein products [8, 22, 31–35]. Model simulation of protein aggregation by accelerated stress methods usually represents a worst-case scenario and results in a polydisperse mixture of protein particles which is difficult to comprehensively characterize and standardize. Most studies are focused on a fraction of aggregates of the certain size range, usually larger than 1 μm, in part due to methodological limitations and method standardization. Much less is known about submicron protein particles and their biological effects. To mimic protein particles of discrete sizes and shapes, we developed a model of protein corona coated engineered nano/micro particles with different core sizes, which results in monodispersed population of particles with defined size distribution, shape, and protein surface characteristics. This model has several limitations, such as the particle shape is the perfect sphere in our study which does not mimic full spectrum of shapes of naturally occurring protein aggregates. We also did not control denaturation status of the corona proteins. Presentation of the corona proteins in different stages of denaturation will be of interest for future studies. Finally, comparison of different particle core materials would be useful for more general conclusions.

Our study, however, demonstrates that the presented model is useful for studying protein particle size- and specific protein- dependent biological effects and may also be useful in future studies of particle shapes and protein surface modifications. We have used HUVEC cell culture as an established EC culture model despite limitation of this specific EC type, related to constitutive and stimulated expression of different surface receptors.

We selected silicon dioxide (SiO_2_) to comprise the core particle. Amorphous silica nano and microparticles are widely used in the biomedical field, can be synthesized in specific monodispersed sizes with tunable porosity at low costs, and have relatively wide ranging biocompatibility [36]. In addition, silica particles are similar by composition to glass nanoparticles and microparticles, important particulate contaminants in biologics [19]. Although silica nanoparticles < 100 nm have been shown cytotoxic to endothelial cells, inducing oxidative stress and apoptosis, toxicity of larger particles is very low [37–39].

Our results did not detect significant effects of 200 nm – 2,000 nm SiMPs on HUVECs viability even at high concentrations (100 µg/ml). We were able to achieve saturated protein binding on the surface of SiMPs, forming a hard protein corona of different immunoglobulin products and HSA for comparison. Interestingly, our CD analysis did not depict dramatic structural changes of immunoglobulin molecules in corona when compared to molecules in solutions. Coronal IgG molecules retained functional characteristics as shown by their ability to bind to Fc receptors and triggering of intracellular pathways.

We have demonstrated that 200 nm IVIG-SiMPs are endocytosed in TNFα-stimulated HUVEC and activate autophagy by an mTOR-dependent pathway. Mechanistic target of rapamycin (mTOR) is a conserved serine/threonine kinase that has a central role in regulation of cell growth and metabolism [40]. mTOR and AMP- activated protein kinase (AMPK) control Ser/Thr kinases ULK1 and ULK2 which in turn control the formation of autophagosomes and autophagic flux. The mTORC1 complex attenuates autophagy by phosphorylating ULK1, and thus inhibition of mTORC1 strongly activates autophagy [40, 41].

The observed cell cycle arrest and apoptosis of HUVECs is in accord with previously described downstream signaling that can occur in mTOR pathways. Interestingly, a significant increase in EV release was seen after IVIG-SiMPs treatment in TNFα-stimulated HUVECs, including EV phenotypes that expressed autophagosome marker LC3 and mitochondrial marker TOM20. A potential link between mTOR activity and EV release has been described, where-mTORC1 inhibition leads to dephosphorylation of N-terminal kinase like protein SCYL1 causing Golgi enlargement, redistribution of early and late endosomes and EV release [42]. Presence of LC3^+^EVs suggests involvement of secretory autophagy pathways including LC3-dependent EV loading and secretion [43].

Our results show that direct toxicity of 200 nm IVIG-SiMPs in TNFα-stimulated HUVECs as well as cellular uptake of the particles can be abolished with anti FcγRII antibody. In concert with this finding, FcγRII were not detected on unstimulated HUVECs but markedly upregulated after TNFα stimulation. A prior study has demonstrated that TNFα and IFNγ significantly increase expression of FcγRII on human aortic endothelial cells [44]. The resistance of unstimulated HUVECs to IVIG-SiMPs toxicity can be explained by lack of sufficient FcγRII expression. The observed toxic effects of IVIG-SiMPs were FcγRII dependent, suggesting that unstimulated HUVECs are resistant to IVIG-SiMPs cytotoxicity.

FcγRs can mediate phagocytosis of large IgG-coated particles as well as pinocytosis of IgG soluble immune complexes (IC) [45–48]. Soluble IC are mainly eliminated by the Kupfer cells and endothelial cells in the liver [47, 49]. Small Ig-IC are endocytosed by clathrin coated pits [47, 50, 51]. Human FcγR consists of three major types, FcγRI (CD64), FcγRII (CD32), and FcγRIII (CD16) [52]. FcγRII has low affinity for monomeric ligand and interacts only with IgG in complex forms. FcγRII is expressed on a wide variety of cell types and frequently represents the sole FcγR class on cells [48, 53]. FcγRII plays an important role in regulation of immune responses, and has three main isoforms FcγRIIA, FcγRIIB and FcγRIIC [54, 55]. FcγRIIA is an activating receptor containing an immunoreceptor tyrosine-based activation motif (ITAM) in the intracytoplasmic domain. Crosslinking of FcγRIIA by IgG results in the phosphorylation of ITAM tyrosine residues, followed by activation of the tyrosine kinase Syk. This leads to calcium mobilization, activation of MAPK pathways and pro-inflammatory cell activation. Interestingly, submicron IVIG-SiMPs augment the proinflammatory phenotype of TNFα-stimulated HUVECs, as manifested by further increase of surface expression of both ICAM-1 and tissue factor. It is unclear the extent to which FcγRIIA signaling is involved in expression of cell surface pro-inflammatory markers. In contrast to FcγRIIA, FcγRIIB is a low affinity inhibitory receptor containing an immunoreceptor tyrosine-based inhibitory motif ITIM in its cytoplasmic tail. FcγRIIB ITIM facilitates negative feedback for signaling of FcγR ITAM coexpressed on the same cell [56, 57]. While the ITIM domain is essential for inhibitory function of FcγRIIB, it is not required for endocytic function of this receptor. FcγRIIB is abundant on liver sinusoidal endothelial cells (LSEC), and it has been shown that this receptor is involved in the clearance of small IC by receptor mediated endocytosis [57–59]. Turman et al. [48] demonstrated FcγRIIB dependent clearance of IgG opsonized HIV particles by LSEC in humanized mice. The size of the cell free HIV immunocomplexes is about 170 nm, which is similar to that of the 200 nm IVIG-SiMPs in our study.

Inhibitory action of FcγRIIB is dependent on the subclass of IgG and the level of FcγRIIB expression. Inhibition of cell activation by the ITIM motif, however, requires co-ligation between the inhibitory and heterologous activating receptors by immune complexes promoting recruitment of inositol phosphatases (SHIP 1/2 or SHP). The phosphatases PTEN and SHIP 1/2 regulate cellular levels of PIP3 by hydrolyzing it to PIP2. Another inhibitory signal may be initiated by ITAM-bearing receptors, like FcγRIIA in the absence of co-ligation with heterologous receptors. This pathway was named as ITAM-mediated inhibitory signal, ITAMi [54]. Monovalent targeting of FcR bearing ITAM motif (e.g., FcγRIIA) induces phosphorylation of the last tyrosine residue of the ITAM motif by Lyn, responsible for transient recruitment of Syk and consequently SHP-1.

A potential pathway to mTOR inhibition is via AMPK which inhibits mTORC1 by phosphorylating regulatory-associated protein of mTOR (RAPTOR) at serine 792 and tuberous sclerosis complex TSC2 at serine 1387 that promotes the inhibitory function of the TSC1-TSC2 complex which controls mTOR activity [40].

Since FcγRIIC is an activating receptor expressed in only 20% of humans, and it’s expression on HUVECs is not known, it is very likely that the mTOR inhibition observed in our study is caused by FcγRIIB ITIM and/or FcγRIIA ITAMi mediated signaling [54, 60]. Based on the observed expression of FcγRII on stimulated HUVECs and the particle size, it is possible that the uptake of 200 nm IVIG-SiMPs in stimulated HUVECs is facilitated by FcγRIIB mediated endocytosis, which is defined as uptake of small (< 0.5 μm) particles without involvement of actin polymerization [48]. Uptake of larger particles, including 1 μm and 2 μm IVIG-SiMPs in our study, is likely mediated by phagocytosis, a different process involving actin polymerization. It is not clear whether mTOR inhibition is directly connected with FcγR signaling or triggered independently after IVIG-SiMPs cellular uptake. Further studies are needed to elucidate the specific mechanism by which IgG/IVIG particles induced mTOR inhibition.

Size-dependent differences in IVIG-SiMP toxicity at the same mass concentration are likely caused by marked difference in the particle surface area and counts, e.g. just based on dimensional calculations, 1000 nm IVIG-SiMPs have 5 x smaller total surface area and about 125 x lower particle count compared to 200 nm IVIG-SiMPs at the same mass concentrations. However, impact of distinct mechanisms of cellular uptake and processing of IVIG-SiMPs of different sizes should also be considered. Further studies are needed to investigate these aspects.

**Fig. 8:**
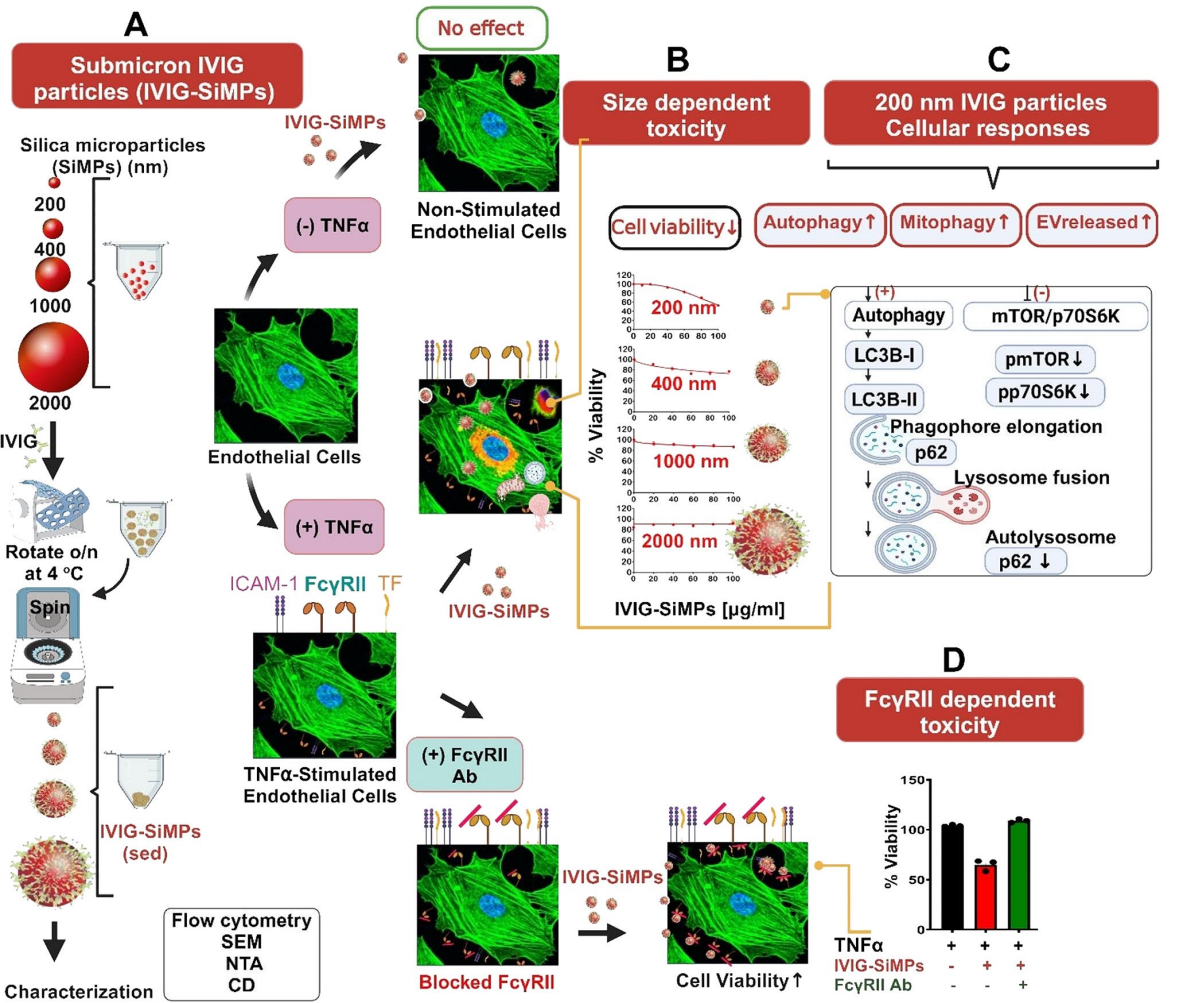
Size-dependent toxicity of submicron immunoglobulin particles on endothelial cells. **(A)** the diagram illustrates the formation of spherical IVIG particles using SiMP core particles, ranging in size from 200 nm to 2 μm. The diagram also highlights several key aspects of submicron IVIG particle toxicity. **(B)** Toxic effects of IVIG-SiMPs were most prominent for 200 nm SiMPs and decreased with larger SiMP size. Interestingly, the toxicity of submicron IVIG-SiMPs only in TNFα-stimulated- but not in unstimulated endothelial cells. **(C)** The effect is associated with multiple cellular responses, including apoptosis induction mTOR-dependent activation of autophagy, mitophagy and the release of extracellular vesicle (EV). Moreover, **(D)** Using blocking antibodies, toxicity of IVIG-SiMPs was found dependent on FcγRII receptor expression on HUVEC, which increased after TNFα-stimulation

In conclusion, as illustrated in Fig. 8, submicron IgG- or IVIG- coated SiMPs decreased viability of proinflammatory stimulated HUVECs in a size-dependent and protein specific manner. The toxicity effect was most prominent in 200 nm SiMPs and decreased with larger SiMP size. 200 nm IVIG-SiMPs in TNFα-stimulated HUVECs caused cell cycle arrest, increase in apoptosis, and mTOR-dependent activation of autophagy. TNFα stimulation caused increase in FcγRII expression on HUVECs. Experiments with FcR blocking antibodies indicate that toxic effects of IVIG-SiMPs are FcγRII dependent.

Our observations are clinically relevant since many patients treated with IVIG are in various stages of an acute or chronic systemic inflammatory response, and we can speculate that their vascular endothelium stimulated by proinflammatory cytokines upregulates expression of FcγRII and may become vulnerable to IgG submicron particle toxicity.

Thus, submicron IgG particles, such as protein aggregates, may impact safety and efficacy of several biologics, including purified immunoglobulin products, plasma derivatives and blood components. Development of standardized and validated analytical methods for characterization and evaluation of biological effects of protein particles of submicron size range is needed. A “two hit” concept (testing effects on stimulated ECs exposed to proinflammatory conditions), should be employed in evaluation of vascular adverse effects of biologics in in vitro, preclinical, and clinical studies. Modelling of protein particles using engineered nanoparticle cores coated with protein corona is a promising approach for investigation of protein particle size/shape dependent biological effects.

### Electronic supplementary material

Below is the link to the electronic supplementary material.

Supplementary Material 1

## Data Availability

The datasets generated during the current study are available from the corresponding author on reasonable request.

## Declarations

### Disclaimer

The findings and conclusions in this article have not been formally disseminated by the Food and Drug Administration and should not be construed to represent any agency determination or policy.
