# Supplementary material for: Submicron immunoglobulin particles exhibit FcγRII-dependent toxicity linked to autophagy in TNFα-stimulated endothelial cells
Source: Cell Mol Life Sci. 2024 Aug 30;81(1):376. doi: 10.1007/s00018-024-05342-9 (PMC11364738; doi:10.1007/s00018-024-05342-9)
Supplement: Supplementary file 1 — Supplementary Material 1 [file 18_2024_5342_MOESM1_ESM.docx]

Submicron immunoglobulin particles exhibit FcγRII-dependent toxicity linked to autophagy in TNFα-stimulated endothelial cells

Wanida C. Hollis^1^, Sehrish Farooq^1^, M. Reza Khoshi^1^, Mehulkumar Patel^1,2^, Elena Karnaukhova^1^, Nancy Eller^1^, Karel Holada^3^, Dorothy E. Scott^1^, Jan Simak^1*^

^1^Center for Biologics Evaluation and Research, FDA, Silver Spring, MD, USA

^2^Center for Devices and Radiological Health, FDA, Silver Spring, MD, USA

^3^Institute of Immunology and Microbiology, First Faculty of Medicine, Charles University, Prague, Czech Republic

***Corresponding author:**

Jan Simak, Ph.D.

Laboratory of Cellular Hematology, Division of Blood Components and Devices, OBRR, Center for Biologics Evaluation and Research, Food and Drug Administration, 10903 New Hampshire Avenue, WO Bldg. 52/72, Rm. 4210, Silver Spring, MD, USA.

Email: [jan.simak@fda.hhs.gov](mailto:jan.simak@fda.hhs.gov)

SUPPLEMENTARY INFORMATION

**ABBREVIATIONS**

| **Abbreviation** |  |
| --- | --- |
| TNFα | Tumor Necrosis Factor-alpha |
| IG | Immunoglobulin |
| Ab | Antibody |
| mAb | Monoclonal antibody |
| IVIG | Intravenous Immunoglobulins |
| IgG | Immunoglobulin G |
| HSA | Human Serum Albumin |
| SiMPs | Silica Microparticles |
| IVIG/HSA-SiMPs | IVIG/HSA corona on SiMPs or IVIG/HSA coated SiMPs |
| HUVECs | Human Umbilical Vein Endothelial Cells |
| SEM | Scanning Electron Microscopy |
| DLS | Dynamic Light Scattering |
| NTA | Nanoparticle Tracking Analysis |
| CD | Circular dichroism |
| CCK-8 | Cell Counting Kit-8 |
| LDH | Lactate Dehydrogenase |
| PI | Propidium Iodide |
| LSCM | Laser Scanning Confocal Microscopy |
| RT | Room temperature |
| WB | Western blot |

MATERIALS AND METHODS

Scanning Electron Microscopy (SEM)

The samples of SiMPs, IVIG-SiMPs, and HSA-SiMPs for SEM were prepared as described in a previously published protocol [59, 60]. 10 µL of each sample were placed on a clean silicon wafer (16006, Ted Pella, Inc.) to dry at room temperature. Dried samples were then coated with 5nm of gold by using EMC Q150R sputter coater. The SEM images were obtained by TESCAN Mira 3 FE-SEM.

Nanoparticle Tracking Analysis (NTA)

Nanoparticle tracking analysis (NTA) was used to evaluate the hydrodynamic diameter size distribution of IVIG-SiMPs particles and the controls SiMPs and HSA-SiMPs. All samples were diluted in PBS. 100 nm silica nanoparticles (DNG-B005, CD Bioparticles) at final dilution of 1:250,000 was used to set up the alignment of ZetaView (Particle Metrix, Germany). Samples were loaded using a 10 ml syringe and filtered 1xPBS was injected between samples to clean the chamber. For each measurement, three cycles were performed by scanning 11 cell positions each and capturing 60 frames per position under following settings: Focus: autofocus; Camera sensitivity for all samples: 80; Shutter: 70; Scattering Intensity: detected automatically; Cell temperature: 28°C. After capture, the videos were analysed by the in-built ZetaView software 8.05.12 SP1 with specific analysis parameters: Maximum area: 2000, Minimum area 10, Minimum brightness: 30. Laser wavelength: 405 nm. camera:0.767 mum/px. Video capturing and data analysis were performed for each sample using the NTA 3.0 software.

Western blot analysis

HUVECs were plated in a 6 well plates at 70-80% confluency and treated with IVIG-SiMPs and the controls SiMPs and HSA-SiMPs in the presence of 10 ng/ml TNF for 24 hrs. Following the incubation, the cells were harvested and resuspended in PBS before subjected to lysis in RIPA buffer supplemented with protease inhibitor cocktail (78429, Thermo Fisher Scientific). The total protein concentration was determined using a bicinchoninic acid (BCA) protein assay kit (23227, Thermo Fisher Scientific). Equal amounts of lysate protein (20 µg) were loaded onto 4-20 % or 12 % Tris-Glycine gel and electrophoretically transferred to nitrocellulose membrane by iBlot2 dry blotting system (IB21001, ThermoFisher Scientific). After blocking with starting blocking buffer (37538, Thermo Fisher Scientific) containing 0.01% Tween-20 for 2 hrs at RT, the membrane was incubated for 2 hrs at RT or overnight at 4^o^C with primary antibodies against autophagic marker such as LC3, p62, mTOR, p70SK, phosphorylated p70SK, compared with β-Actin, and/or beta-tubulin. The immunoreactive bands were visualized by incubation with horseradish peroxidase (HRP)-conjugated anti rabbit or mouse immunoglobulin G (1:5000) at RT for 2 hrs. Peroxidase activity was visualized with Super Signal West Fem to maximum sensitivity substrate (34094, Thermo Fisher Scientific) and visualized by gel documentation, Syngene (Synoptics Ltd). The bands were quantified by ImageJ software [28].

Detection of FcγR on TNFα stimulated HUVECs (ELISA)

HUVECs were cultured with varying concentrations of TNFα (0, 5, 10, 20 ng/ml) for 24 hrs. Following the treatment, cells were fixed with 2% paraformaldehyde (PFA) for 10 mins at RT to preserve cellular structure. Subsequently, the cells were blocked with 2% bovine serum albumin (BSA) at 37°C for 1 hr followed by incubation for 2 hrs with 5 μg/ml of anti FcγR α-HU CD64, α -HU CD32, α -HU CD16 and isotype control (all obtained from Thermo Fisher Scientific). After three washes with PBS, optimum concentrations of anti-mouse IgG labelled with HRP were added and incubated for an additional 2 hrs. After several washings, 3,3',5,5'-Tetramethylbenzidine (TMB) was added and incubated for 15 mins to initiate a colorimetric reaction. The reaction was then stopped with 4 N H_2_SO_4_. The bound enzyme activity was measured spectrophotometrically at 450 nm using a microtiter plate reader.

**Suplementary Table T1. Hydrodynamic size range of bare SiMPs, IVIG-SiMPs, and HSA-SiMPs assessed by NTA.**

|  |  |  |  |  |
| --- | --- | --- | --- | --- |
| Sample | Diameter [nm] | Particles/ml | FWHM^1^ | Percentage |
| **SiMPs**  Population 1  Population 2 | 195.2  72.3 | 3.3 x 10^6^  3.5 x 10^5^ | 149.7  10.5 | 94.8%  5.2% |
| **IVIG-SiMPs**  Population 1  Population 2  Population 3 | 214.0  285.2  173.3 | 9.6 x 10^5^  8.2 x 10^5^  8.0 x 10^5^ | 44.9  50.4  41.7 | 47.2%  29.2%  23.6% |
| **HSA-SiMPs**  Population 1  Population 2  Population 3 | 253.3  322.2  134.0 | 2.5 x10^6^  1.6 x10^6^  2.4 x10^5^ | 92.9  60.6  18.5 | 65.8%  28.7%  5.5% |

^1^FWHM– full width at half maximum intensity.

**Supplementary Table T2. Percentage of the secondary structure elements of IgG-SiMPs and HSA-SiMPs and their controls.**

|  |  |  |  |  |  |  |
| --- | --- | --- | --- | --- | --- | --- |
| Sample | H(r) | H(d) | S(r) | S(d) | Turns | Unordered |
| IgG-SiMPs | 0.3 | 3.2 | 29 | 13.8 | 21.4 | 32.3 |
| IgG ctrl | 0.1 | 3.2 | 29.3 | 13.9 | 21.2 | 32.3 |
| HSA-SiMPs | 36.8 | 20.9 | 0 | 5.9 | 16.7 | 19.7 |
| HSA ctrl | 31.4 | 23.6 | 0.1 | 7 | 18.1 | 19.6 |

H(r) – regular a-helix; H(d) – a-distorted helix; S(r) – regular β-sheet/strand; S(d) – distorted β-sheet/strand; ctrl – control.

FIGURES AND LEGENDS


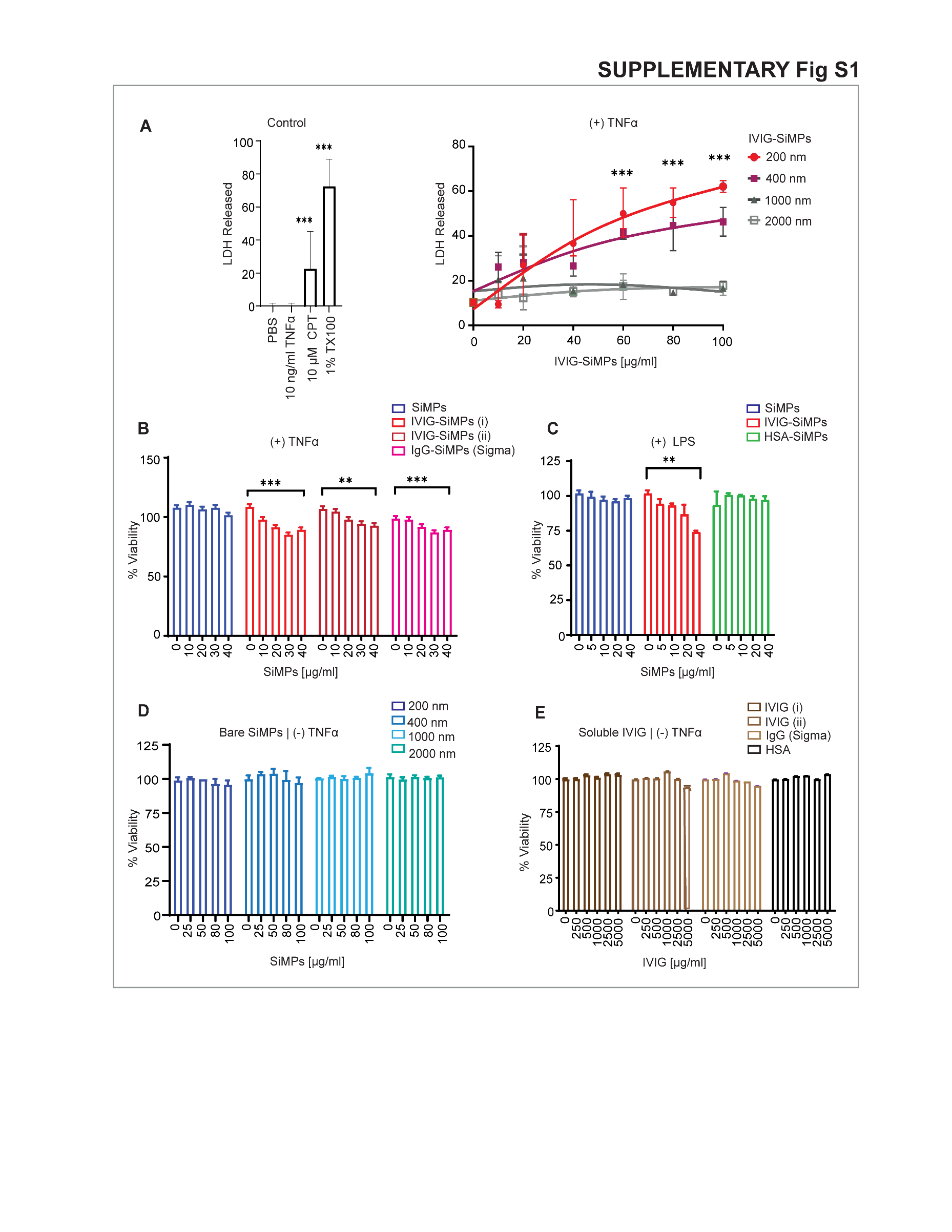


**Supplementary Fig. S1.** **(A)** Dose-response toxicity of different sizes of IVIG-SiMPs in TNFα-stimulated HUVEC after 24 hrs treatment assessed by LDH assay. 10 µM CPT and 1% TX100 were used as positive controls. Viability (CCK-8) of **(B)** 10 ng/ml TNFα- and **(C)** 2 µg/ml LPS-stimulated HUVECs after 24 hrs treatment with 200 nm SiMPs (100 µg/ml) coated with IVIG and HSA. Cytotoxicity screening of **(D)** bare SiMPs (0-100 µg/ml) and **(E)** soluble IVIG (0-5 mg/ml) in unstimulated HUVECs. Mean ± SEM, n = 3, ** p < 0.01, *** p < 0.001.


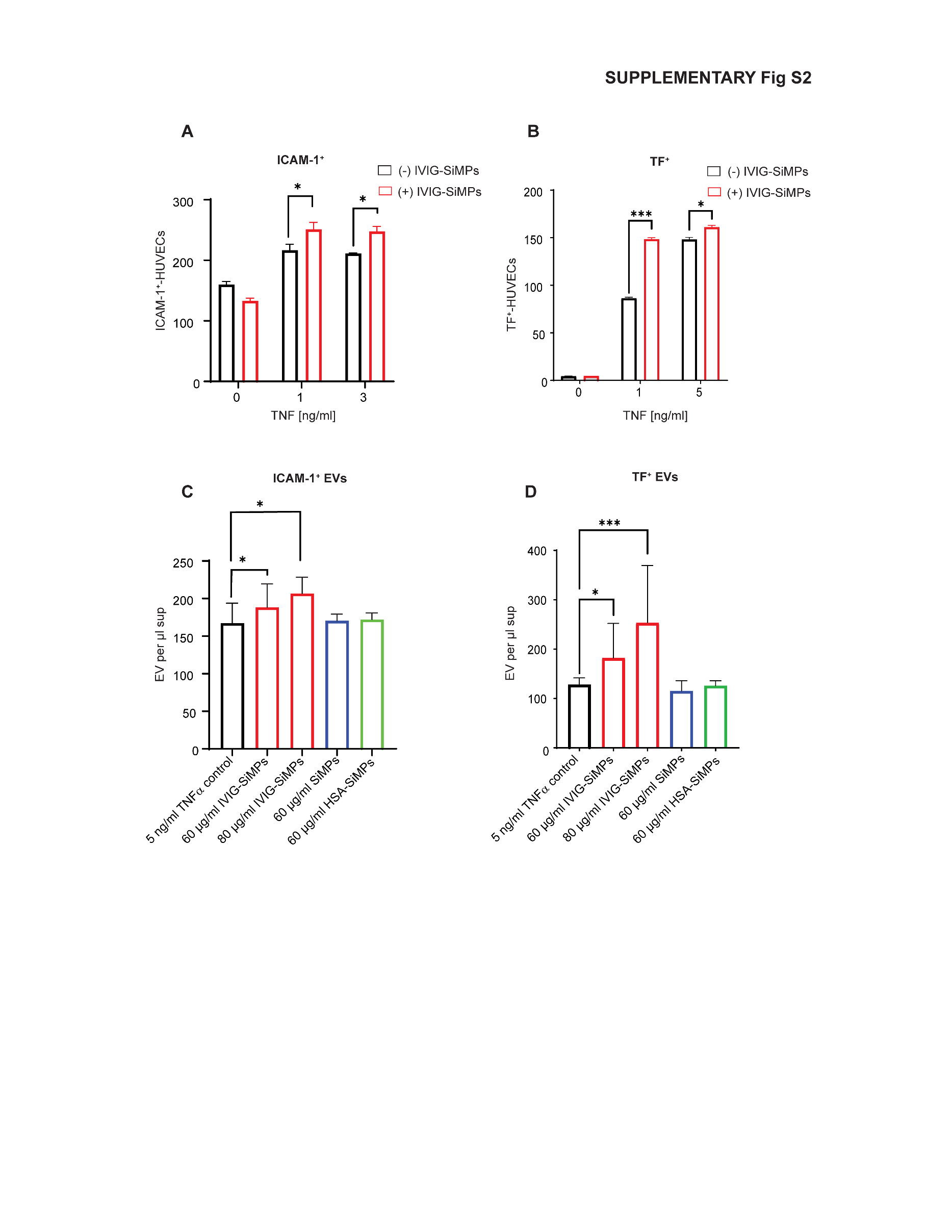


**Supplementary Fig. S2.** Analysis of ICAM1 and TF expression in TNFα-stimulated HUVECs treated with IVIG-SiMPs. **(A)** Flow cytometry analysis demonstrated enhanced ICAM1 expression and **(C)** EVs secretion following exposure to IVIG-SiMPs in TNFα-stimulated HUVECs for 24 hrs. A similar pattern was observed in PE-conjugated TF staining; IVIG-SiMPs augmented **(B)** TF expression levels (PE-TF^+^) and **(D)** EVs release in HUVECs stimulated with 1 and 5 ng/ml TNFα. Mean ± SEM, n = 3, * p< 0.05, ** p < 0.01, *** p < 0.001


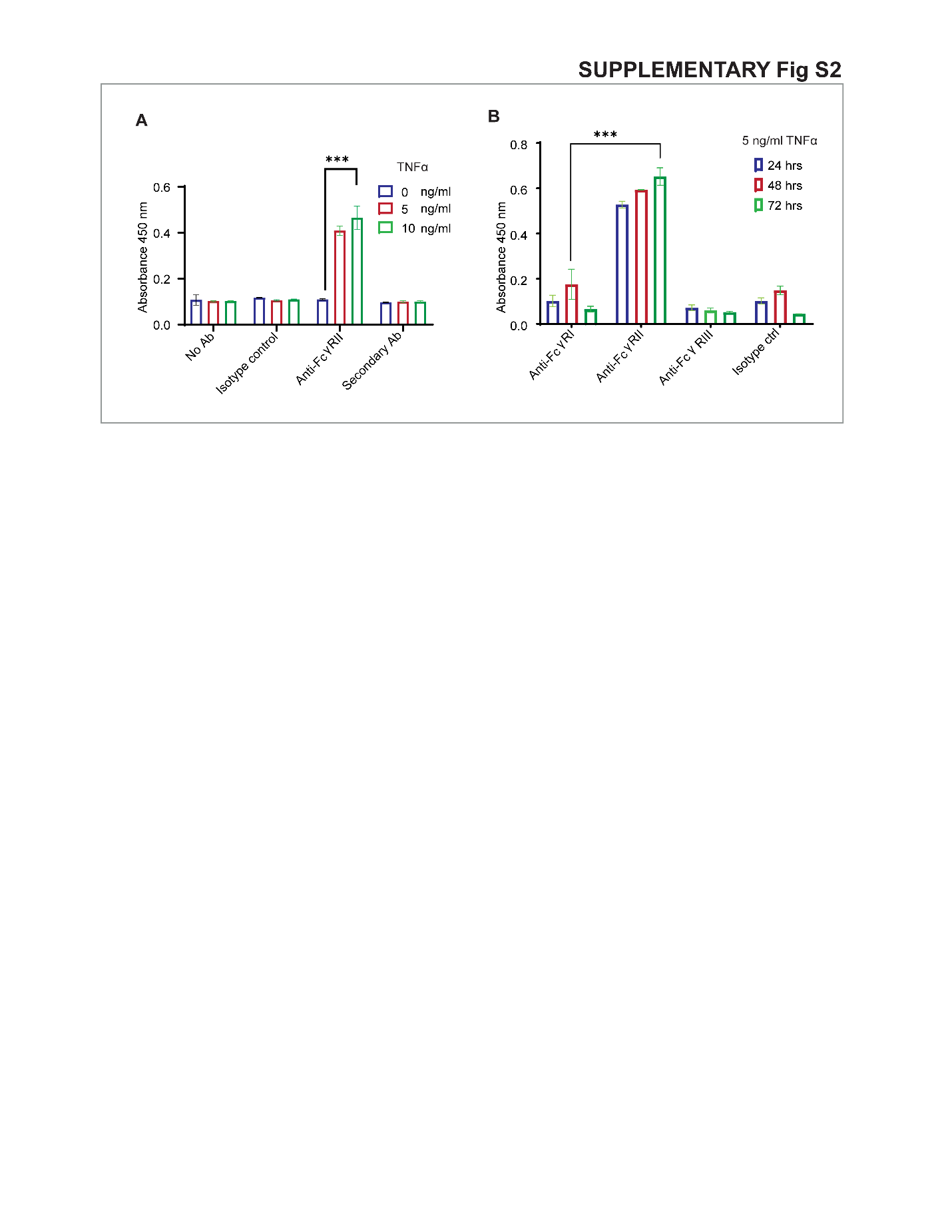


**Supplementary Fig. S3.** The presence of FcγRs in TNFα-stimulated HUVECs was assessed using In cell ELISA. **(A)** HUVECs were stimulated with different concentrations of TNFα (ranging from 0 to 10 ng/ml) for 24 hrs. **(B)** HUVECs were stimulated with 10 ng/ml TNFα for 24, 48, and 72 hrs before undergoing ELISA analysis. Mean ± SEM, n = 3, *** p < 0.001.
